# Paracoccidioidomycosis: an uncommon clinical presentation^⋆^^⋆⋆^

**DOI:** 10.1016/j.abd.2020.06.001

**Published:** 2020-08-29

**Authors:** Bruno Augusto Alvares, Cláudia Alves Lapa Gracia, Mariangela Esther Alencar Marques, Silvio Alencar Marques

**Affiliations:** a Department of Dermatology and Radiotherapy, Faculdade de Medicina, Universidade Estadual Paulista, Botucatu, SP, Brazil; b Private Clinic, Birigui, SP, Brazil

**Keywords:** Aged, Diagnosis, Paracoccidioidomycosis, Skin and connective tissue diseases, Treatment outcome

## Abstract

Paracoccidoiomycosis is a systemic mycosis with a higher incidence in males with history of exposure to the rural environment; its classic clinical manifestation is an oro-pulmonary lesion. The authors report a case of a female, urban, 76-year-old patient with atypical clinical-dermatological presentation and diagnostic conclusion after histopathological examination. The clinical response was quick and complete after treatment with itraconazole 400 mg/day in the first month, decreased to 200 mg/day until the sixth month of treatment.

Paracoccidioidomycosis is a systemic mycosis caused by dimorphic fungi of the genus *Paracoccidioides* (*P. brasiliensis* or *P. lutzii*),^1^ which, in the chronic clinical presentation in adults, mainly affects men (male/female ratio: up to 22/1) in the range from 30 to 59 years old.^2^ Lung and oral mucosa involvement is usual in this clinical form.

The authors report a case of a 76-year-old female patient with painful skin lesions two months prior to presentation. The patient denied trauma or fever; she had undergone treatment with antibiotics, without improvement. She reported type II diabetes mellitus and systemic arterial hypertension. The dermatological examination showed phagedenic ulcers, with necrotic areas, surrounded by an inflammatory halo (Figure 1, Figure 2). The lesions were more exuberant in the deltoid regions, with satellite lesions on the shoulders and forearms. The supplemental clinical examination was normal, although the patient presented depression symptoms and was suffering from her illness.

**Figure 1:**
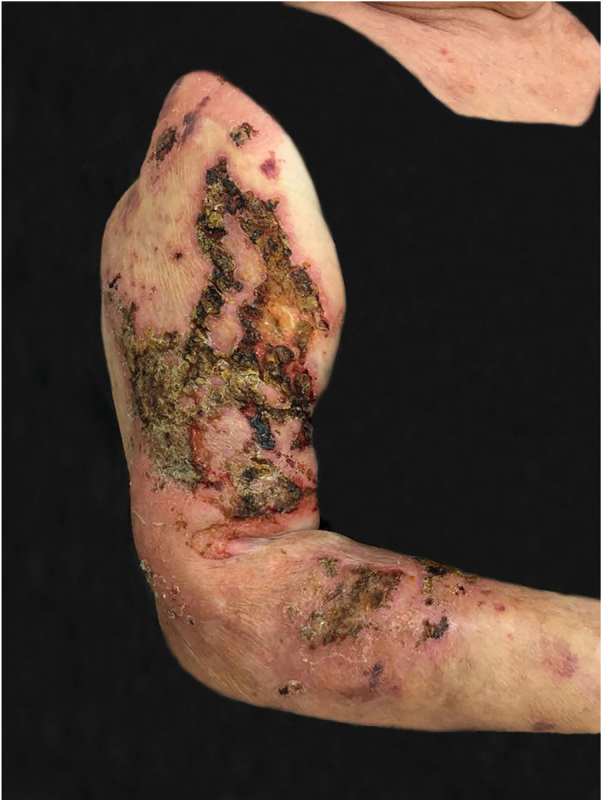
Paracoccidioidomycosis: ulcerative-necrotic, phagedenic lesions, with areas covered by crusts, affecting the right arm and forearm.

**Figure 2:**
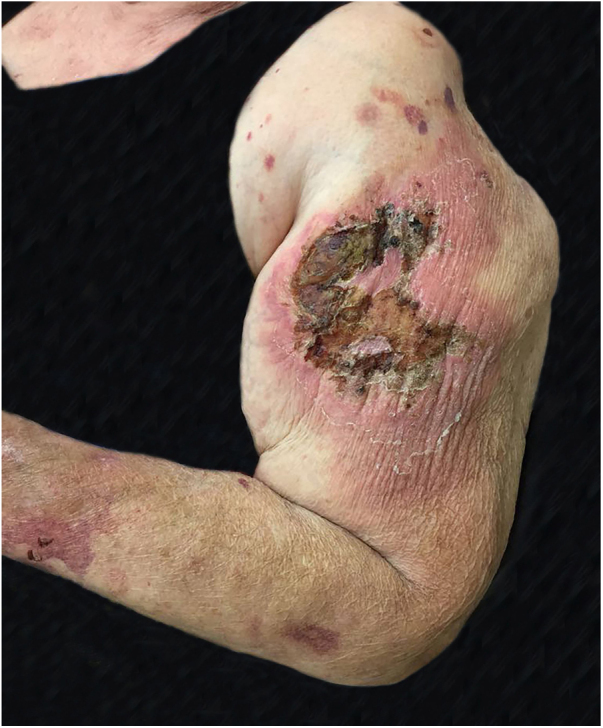
Paracoccidioidomycosis: extensive ulcerative necrotic lesion, areas covered by crusts and with an intense inflammatory halo. Papules and satellite plaques, located on the left arm and forearm.

The clinical hypothesis was that of pyoderma gangrenosum, due to the presence of painful ulcers, of rapid growth and geographical aspect; however, no violet halo was observed and the edges were not undermined. Another hypothesis was primary cutaneous cryptococcosis, due to the inflammatory aspect associated with necrosis, localized in exposed areas; however, the bilateral aspect of the lesions did not correspond to this hypothesis. Biopsies and laboratory investigation were performed; the final diagnosis of paracoccidioidomycosis was surprising and confirmed by histopathological examination (Figure 3, Figure 4) and direct mycological examination. Complementary tests, including chest and abdominal computed tomography (CT), ruled out involvement of other organs. Laboratory tests revealed elevated CRP and glycemia, negative HIV and anti-*P. brasiliensis* serology, and negative culture for bacteria and fungi. The clinical response was complete after treatment with itraconazole 400 mg/day in the first month, decreased to 200 mg/day until the sixth month of treatment.

**Figure 3:**
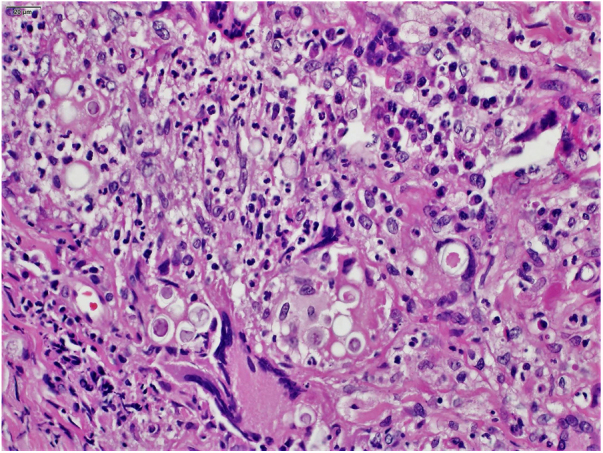
Paracoccidioidomycosis: chronic granulomatous inflammatory infiltrate with the presence of fungal cells within the cytoplasm of giant cells and macrophages. (Hematoxylin & eosin, ×400).

**Figure 4:**
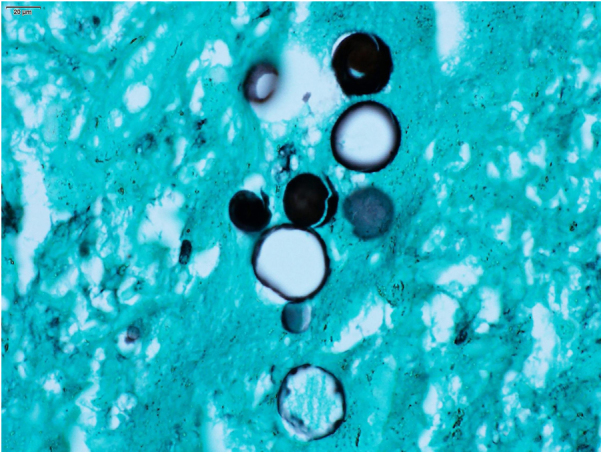
Paracoccidioidomycosis: *Paracoccidioidesspp*. Fungal cells, resembling a Mickey Mouse head. (Grocott-Gomori, immersion, 1000×).

The present case differs from the usual pattern of paracoccidioidomycosis in several aspects. The disease is uncommon in both female and elderly patients. As a rule, lung involvement is present in adult patients, being the source of metastatic spread of the infection to other organs.^3^ Pulmonary involvement was not observed in the present case, even using high-resolution CT.^4^ Nonetheless, the most unusual finding was the pattern of skin lesions. In paracoccidioidomycosis, skin lesions are observed in up to 62.1% of cases, usually on the face, such as acneiform lesions, in infiltrated plaques, or vegetating and simultaneously with multiple organ involvement, including the lungs.^5^ Ulcers with necrotic areas are uncommon, as the skin ulcers in paracoccidioidomycosis are generally shallow, granular, and usually with hemorrhagic spots, similar to what is observed in mucosal lesions; they are not phagedenic or painful.^5^

The patient's advanced age, immunosenescence, and diabetes probably contributed to the rapid evolution and atypical presentation in this case.^6^, ^7^ Itraconazole has been considered as the first treatment option and is effective at a dose of 200 mg/day for 9–18 months.^8^ In the present study, itraconazole was successfully used at 400 mg/day in the first month with the aim of promoting quick resolution of injuries, improving quality of life, and reducing the psychological stress experienced by the patient.

## Financial support

None declared.

## Authors’ contribution

Bruno Augusto Alvares: Approval of the final version of the manuscript; conception and planning of the study; intellectual participation in propaedeutic and/or therapeutic conduct of studied cases.

Cláudia Alves Lapa Gracia: Approval of the final version of the manuscript; intellectual participation in propaedeutic and/or therapeutic conduct of studied cases; critical review of the manuscript.

Mariangela Esther Alencar Marques: Approval of the final version of the manuscript; intellectual participation in propaedeutic and/or therapeutic conduct of studied cases; critical review of the manuscript.

Silvio Alencar Marques: Approval of the final version of the manuscript; conception and planning of the study; critical review of the manuscript.

## Conflicts of interest

None declared.
